# Genetic Variants in Human Leukocyte Antigen-*DP* Influence Both Hepatitis C Virus Persistence and Hepatitis C Virus F Protein Generation in the Chinese Han Population

**DOI:** 10.3390/ijms15069826

**Published:** 2014-06-03

**Authors:** Xiaodong Xu, Ming Yue, Longfeng Jiang, Xiaozhao Deng, Yongxiang Zhang, Yun Zhang, Danyan Zhu, Wen Xiao, Zhenxian Zhou, Wenjuan Yao, Jing Kong, Xiaojie Yu, Juan Wei

**Affiliations:** 1 Department of Biochemistry and Molecular Biology, School of Basic Medicine, Nanjing Medical University, Nanjing 210029, China; E-Mails: dongxiaoxuok@163.com (X.X.); zhudanyan1907@126.com (D.Z.); yuxiaojie0903@126.com (X.Y.); juanwei_315@163.com (J.W.); 2 National Clinical Research Center of Kidney Diseases, Jinling Hospital, Nanjing University Clinical School of Medicine, Nanjing 210002, China; 3 School of Life Science and Technology, China Pharmaceutical University, Nanjing 210009, China; E-Mails: njym08@163.com (M.Y.); xiaowen2013cpu@163.com (W.X.); 4 Department of Infectious Diseases, the First Affiliated Hospital with Nanjing Medical University, Nanjing 210029, China; E-Mail: phoenix@126.com; 5 Institute of Disease Control and Prevention, Huadong Research Institute for Medicine and Biotechnics, Nanjing 210002, China; 6 Department of Clinical Laboratory, Nanjing Second Hospital, Nanjing 210003, China; E-Mail: zzx0417@126.com; 7 Department of Pharmacology, Nantong University Medical College, Nantong 226019, China; E-Mail: juanwenyy@ntu.edu.cn; 8 School of Life Science and Chemical Engineering, Huaiyin Institute of Technology, Huaian 223003, China; E-Mail: cpukj@163.com

**Keywords:** F protein, hepatitis C virus, human leukocyte antigen-*DP*, polymorphism

## Abstract

Chronic hepatitis C is a serious liver disease that often results in cirrhosis or hepatocellular carcinoma. The aim of this study was to assess the association of *human leukocyte antigen-DP* (*HLA-DP*) variants with risk of chronic hepatitis C virus (HCV) or anti-F antibody generation. We selected two single nucleotide polymorphisms (SNPs) in a region including *HLA-DPA1* (rs3077) and *HLA-DPB1* (rs9277534) and genotyped SNPs in 702 cases and 342 healthy controls from the Chinese population using TaqMan SNP genotyping assay. Moreover, the exon 2 of the *HLA-DPA1* and *HLA-DPB1* genes were amplified and determined by sequencing-based typing (SBT). The results showed that rs3077 significantly increased the risk of chronic HCV infection in additive models and dominant models (odds ratio (OR) = 1.32 and 1.53). The rs3077 also contributed to decrease the risk of anti-F antibody generation in additive models and dominant models (OR = 0.46 and 0.56). Subsequent analyses revealed the risk haplotypes (DPA1*0103-DPB1*0501 and DPA1*0103-DPB1*0201) and protective haplotypes (DPA1*0202-DPB1*0501 and DPA1*0202-DPB1*0202) to chronic HCV infection. Moreover, we also found that the haplotype of DPA1*0103-DPB1*0201 and DPA1*0202-DPB1*0202 were associated with the anti-F antibody generation. Our findings show that genetic variants in *HLA-DP* gene are associated with chronic HCV infection and anti-F antibody generation.

## 1. Introduction

Chronic infection with hepatitis C virus (HCV) is a serious liver disease worldwide that frequently results in cirrhosis or hepatocellular carcinoma (HCC) within two to three decades of infection. It is estimated that more than 180 million people have been infected with HCV worldwide, and approximately 3–4 million new cases are considered to be infected by HCV [1]. However, only 10%–20% patients with acute infection clear the virus spontaneously, while the remainder progress to chronic HCV infection and are at risk of cirrhosis and HCC. Moreover, researchers from the Centers for Disease Control and Prevention (CDC) in the United States have determined that the number of deaths related to hepatitis C have exceeded the number related to human immunodeficiency virus (HIV) [2].

The mechanisms of chronic HCV infection and factors influencing the disease progression are still not fully understood, but several epidemiological factors such as virus variants, infection period, gender, treatment, host genetic variation and environment are suspected to affect the clinical outcomes [3,4]. For example, as the host genetic variation, it is well establishments that the single nucleotide polymorphisms (SNPs) of rs8099917 and rs12979860 located near the *interleukin-28B* (*IL-28B*) are strongly associated with a virological response to pegylated interferon-α/ribavirin (PEG-IFNα/RBV) combination therapy and spontaneous HCV clearance [5,6,7,8,9]. Aspects of the virus itself, as we all know, such as the HCV core protein (Core) encoded by the viral genome plays a vital role in the virus survival and pathogenicity, including virion assembly, influence of cytokine generation, promotion or repression of apoptosis and cellular immune response [10,11,12]. Recently, a “new Core” protein synthesized by a genome open reading frame (ORF) shift was named alternative reading frame protein (F protein) [13,14]. It has been demonstrated that the humoral and cellular immune responses to HCV F protein in the sera of HCV-infected patients were detected, and the rate of F-seropositivity was increased along with the progression of hepatitis C [15,16,17]. Other studies also revealed that the F protein has significant effects not only on the inhibited expression of p21 but also on promoting the development of chronic hepatitis by disturbing the balance of cytokine secretion [15,18]. All these data implied that the expression of the F protein during HCV infection plays an important role in the development of persistent infection. However, HCV F protein generation in host after virus infection varies enormously among individuals. The reason for HCV F protein generation in subjects after HCV infection remains unclear.

The *human leukocyte antigen* (*HLA*) II class molecules process and present viral antigen polypeptide to CD^+^4 T helper cells and lead to an immune response to viral invasion, and our previous research also found that F protein expression was related with the Th2 biased cytokine response in chronic hepatitis C patients [17]. Thus, the *HLA* molecules and T cell helper response is crucial in HCV progression. Moreover, the polymorphism in the *HLA* loci affects the spectrum of viral peptides and the contributions to the clinical outcomes of HCV infection has been identified in several studies. HLA-DR*13, HLA-DRB1*11 and HLA-DQB1*0301 alleles were significantly protective against HCV infection, but the polymorphism of codon 145 Gln > Lys in the *LMP7* gene is significantly associated with HCV persistence [19,20,21]. To date, *HLA-DP* genes have been somewhat neglected in relation to their impact on human disease when compared with other loci of *HLA*, partly because *HLA-DPA1* and *HLA-DPB1* polymorphisms do not vary greatly. Another reason is the level of *HLA-DP* molecular expression on the cell surface are likely to be lower than that of *HLA-DR* or *HLA-DQ* [22]. Recently, Kamatani *et al.* conducted a two-stage genome-wide association study (GWAS) and identified two SNPs in *HLA-DPA1* (rs3077) and *HLA-DPB1* (rs9277535) that were associated with persistent hepatitis B virus (HBV) infection in Asians [23]. Subsequently, the effects of rs3077 and rs9277535 have also been verified in other populations and diseases, including chronic berylliasis, juvenile rheumatoid arthritis and cervical cancer [24,25,26]. In addition, Rasmi *et al.* also identified a novel variant (rs9277534) in the 3'-untranslated regions (UTR) of *HLA-DPB1* loci, which was significantly associated with HBV recovery in several populations [27]. Importantly, the rs9277534, unlike the rs9277535, could distinguish the most protective allele (DPB1*0401) from the susceptible allele (DPB1*0101) [27]. The variants of the *HLA-DP* gene (rs3077 and rs9277534) not only confers significant association with HBV persistence, but also changes the levels of *HLA-DP* surface protein or transcript level expression [23,27]. The molecules encoded by *HLA-DP* genes are expressed on the surface of antigen-presenting cells (APC), and interact with both the peptides and the CD4^+^ T-helper lymphocytes receptors; the polymorphisms in these genes might result in amino acid substitutions in the *HLA-DP* molecules or changes in gene regulation. This evidence indicates that the variants of the *HLA-DP* may play an important role in disease progression and recovery.

Because clinical outcomes after exposure to HCV are highly variable, identification of genetic and viral factors that are related to chronic HCV is critical. In 2013, we investigated the association between HCV F protein and *HLA-DR* or *HLA-DQ* alleles in HCV infected patients in a small size [17]. In view of HCV F protein playing a special role in the development of persistent infection and *HLA-DP* function in presenting extracellular antigens to CD4^+^ T cells, it is necessary to validate the association between the variants in *HLA-DP* locus and the risk of chronic HCV and HCV F protein generation. Moreover, studies about variants within the *HLA-DP* locus with chronic HCV infection and HCV F protein are sparse. The study in this area might explain a possible new vision of the development of chronic HCV. To evaluate the issues outlined above, we selected the most strongly associated SNPs of rs3077 in *HLA-DPA1*, rs9277534 in *HLA-DPB1* and genotyped these two polymorphisms in a case-control study of Chinese Hans from Jiangsu Province.

## 2. Results and Discussion

### 2.1. Result

#### 2.1.1. Study Population’s Characteristics

Characteristics of 342 healthy controls, 186 F-seronegative patients and 516 subjects with F-seropositivity were shown in Table 1. No significant sex differences among the three groups (*p* = 0.057), and also no statistical difference existed in terms of HCV RNA level and HCV genotypes between the group of HCV F-seronegative patients and the group of F-seropositivity (*p* = 0.925; *p* = 0.077). The results are consistent with our previous research and others reports [15,17,28]. However, subjects were significantly older in the group of F-seropositive subjects than in the group of F-seronegative patients and healthy control (*p* = 0.003). Moreover, compared to the healthy controls and the group of HCV F-seronegative patients, the group of F-seropositive patients had higher levels of ALT/AST and lower numbers of platelets (*p* < 0.001, respectively). Notably, with the development of liver fibrosis, the percentage of F-seropositive patients was increased (*p* < 0.001).

#### 2.1.2. Relationship between *Human Leukocyte Antigen-DP* (*HLA-DP*) Gene Polymorphisms and Chronic Hepatitis C Virus (HCV) Infection or Anti-F Antibody Generation

The genotype distributions of rs3077 (*HLA-DPA1*) and rs9277534 (*HLA-DPB1*) among the three groups were described in Table 2. The observed genotype frequencies of the two SNPs in Hardy-Weinberg equilibrium among the 342 healthy controls, 186 F-seronegative patients and 516 F-seropositive subjects (Control: rs3077, *p* = 0.08, rs9277534: *p* = 0.06; F-seronegative group: rs3077, *p* = 0.302, rs9277534: *p* = 0.767; F-seropositive group: rs3077, *p* = 0.911, rs9277534: *p* = 0.805; respectively). We conducted logistic regression analyses with adjustment for age, sex and/or HCV genotype. In Table 2, the results of association analysis were represented with the rs3077 variant genotypes significantly increased for the chronic HCV risk in additive genetic models (adjusted odds ratio (OR) = 1.32, 95% confidence interval (CI) = 1.08–1.60) and dominant genetic models (adjusted OR = 1.53, 95% CI = 1.18–1.98). But no evidence showed significant associations between the genotypes of rs9277534 and the risk of chronic HCV. In addition, rs3077 variant genotypes significantly decreased the risk of anti-F antibody generation, when F-seropositive patients were compared with F-seronegative patients in additive genetic models (adjusted OR = 0.56, 95% CI = 0.44–0.72), and dominant genetic models of rs3077 alleles (adjusted OR = 0.46, 95% CI = 0.32–0.66).

**Table 1:** Distributions of selected variables in HCV-infection patients and healthy controls.

Variables	Controls	Cases		*p*
	Healthy Controls *n* = 342 (%)	Anti-F Negative *n* = 186 (%)	Anti-F Positive *n* = 516 (%)	
Age, year	56.97 ± 8.34	55.35 ± 7.46	57.61 ± 7.58	0.003 *
Sex				0.057 *
Females	228 (66.7)	142 (76.3)	352 (68.2)	
Males	114 (33.3)	44 (23.7)	164 (31.9)	
ALT (UL)	22.83 ± 7.36	26.05 ± 14.02	59.04 ± 43.06	<0.001 *
AST (UL)	24.56 ± 6.65	29.61 ± 17.02	54.14 ± 37.16	<0.001 *
PLT count (×10^9^/L)	194.25 ± 39.42	182.21 ± 45.93	168.64 ± 51.47	<0.001 *
HCV RNA (×10^6^ copies/mL)	–	3.19 ± 2.72	3.04 ± 3.52	0.925 **
HCV genotype				0.077 **
1a	–	48 (25.9)	129 (25.0)	
1b	–	94 (50.5)	301 (58.3)	
Non-1		44 (23.7)	86 (16.7)	
Stage of liver fibrosis				<0.001 ^#^
F0	–	20 (10.8)	19 (3.7)	
F1	–	108 (58.1)	229 (44.4)	
F2	–	29 (15.6)	96 (18.6)	
F3	–	20 (10.8)	96 (18.6)	
F4	–	9 (4.8)	76 (14.7)	

Data were expressed as mean ± standard deviation. Abbreviation: HCV, hepatitis C virus; ALT, alanine aminotransferase; AST, aspartate aminotransferase; PLT, platelet; F0, no fibrosis; F1, mild fibrosis; F2, moderate fibrosis; F3, severe fibrosis; F4, cirrhosis; * *p* values were given by chi-square among the group of controls and cases; ** *p* values were given by chi-square between the group of anti-F negative and anti-F positive; ^#^ *p* value was given by Mantel-Haenszel method.

Subsequently, we evaluated combined effects of *HLA-DPA1* rs3077 and *HLA-DPB1* rs9277534 by adding up the number of variant alleles of the independent SNPs on anti-F antibody generation risk. In Table 3, there was significant difference in combined rs3077 and rs9277534 between the F-seropositive group and F-seronegative group. The results showed that the ORs for the risk of anti-F antibody generation was significantly decreased with the increasing number of variants alleles of the two SNPs in a dose-dependent manner (*p* for trend = 0.001). Compared with subjects without any variant allele of the two SNPs, subjects carrying one to four variant alleles of rs3077 and rs9277534 had significant association with HCV F-seronegative (OR = 0.52, 95% CI = 0.33–0.84).

#### 2.1.3. Results of the Haplotype Analysis

Pairwise linkage disequilibrium (LD) of these two SNPs was calculated by both *D*’ and *r*^2^, no significant LD was observed between these two SNPs. To further assess the effect of variant alleles of *HLA-DP* on chronic HCV and anti-F antibody generation (Table 4), we constructed the haplotype analysis and found the haplotype of T–A had a 1.08 fold risk of chronic HCV (OR = 2.08, 95% CI = 1.25–3.45). Moreover, the haplotype of T–A and T–G showed a significant lower risk of anti-F antibody generation (T–A: OR = 0.44, 95% CI = 0.27–0.71; T–G: OR = 0.59, 95% CI = 0.45–0.77; respectively) when compared with the most frequent C–A haplotype, which was consistent with the single SNP analysis.

**Table 2:** Genotype distribution and association of rs3077 and rs9277534 with risk of chronic HCV infection and anti-F antibody generation.

SNP	Anti-F Positive *n* = 516 (%)	Anti-F Negative *n* = 186 (%)	Healthy Controls *n* = 342 (%)	Cases ^†^ *vs.* Healthy Controls		Anti-F Positive *vs.* Anti-F Negative	
				OR (95% CI)	*p* *	OR (95% CI)	*p* *
rs3077							
CC	243 (47.1)	53 (28.5)	180 (52.6)	–	–	–	–
CT	223 (43.2)	99 (53.2)	127 (37.1)	**1.55 (1.17–2.04)**	**0.002**	**0.51 (0.35–0.75)**	**<0.001**
TT	50 (9.7)	34 (18.3)	35 (10.2)	1.45 (0.94–2.24)	0.095	**0.33 (0.20–0.55)**	**<0.001**
additive model				**1.32 (1.08–1.60)**	**0.006**	**0.56 (0.44–0.72)**	**<0.001**
dominant model				**1.53 (1.18–1.98)**	**0.001**	**0.46 (0.32–0.66)**	**<0.001**
rs9277534							
AA	169 (32.8)	47 (25.3)	114 (33.3)	–	–	–	–
AG	255 (49.4)	95 (51.1)	152 (44.4)	1.22 (0.90–1.64)	0.198	0.77 (0.52–1.16)	0.194
GG	92 (17.8)	44 (23.7)	76 (22.2)	0.94 (0.66–1.36)	0.752	0.62 (0.38–0.99)	0.047
additive model				0.99 (0.83–1.19)	0.947	0.80 (0.62–0.99)	0.044
dominant model				1.13 (0.85–1.48)	0.404	0.73 (0.50–1.07)	0.088

Abbreviation: OR, odds ratio; CI, confidence interval; Dominant model: (wild homozygote)/(heterozygote + variant homozygote); Additive model: (minor homozygote *vs.* heterozygote *vs.* major homozygote); * *p* values are two sided and were calculated by logistic regression analyses adjusted for sex, age and/or HCV genotype; **^†^** Cases including the group of anti-F positive and anti-F negative. Multiple testing: using Bonferroni adjustment. Significance levels after Bonferroni correction for multiple testing were *p* = 0.025 (0.05/2); Bold represent the *p* values were considered statistically significant.

**Table 3:** Cumulative effects on combined variant alleles (rs3077-T and rs9277534-G) with risk of anti-F antibody generation.

Number of Risk Alleles ^#^	Anti-F Positive *n* = 516 (%)	Anti-F Negative *n* = 186 (%)	Anti-F Positive *vs.* Anti-F Negative	
			OR (95% CI)	*p* *
0	137 (26.6)	29 (15.6)	–	–
1	117 (22.7)	33 (17.7)	0.75 (0.42–1.39)	0.271
2	174 (33.7)	75 (40.3)	**0.52 (0.32–0.85)**	**0.007**
3–4	88 (17.1)	49 (26.3)	**0.38 (0.23–0.69)**	<0.001
Trend (*p*)				0.001 **
0	137 (26.6)	29 (15.6)	–	–
1–4	379 (73.4)	157 (84.4)	**0.52 (0.33–0.84)**	**0.004**

Abbreviation: OR, odds ratio; CI, confidence interval; ^#^ rs3077-T and rs9277534-G were assumed as risk alleles; * *p* value was given by logistic regression analyses adjusted for sex, age and/or HCV genotype; ** *p* value was given by 2 × 4 table chi-square; Bold represent the *p* values were considered statistically significant.

#### 2.1.4. Association of *HLA-DP* Alleles with Chronic HCV Infection and Anti-F Antibody Generation

Subsequently, in order to investigate the association of *HLA-DP* alleles with chronic HCV infection and anti-F antibody generation risk, we genotyped *HLA-DPA1* and *HLA-DPB1* alleles by direct sequencing of exon 2 and found DPA1*0103 and DPB1*0201 increased the chronic HCV infection risk (OR = 2.45, 95% CI = 2.0–3.0; OR = 1.66, 95% CI = 1.28–2.14; respectively), whereas DPA1*0202 and DPB1*0202 decreased the chronic HCV infection risk (OR = 0.79, 95% CI = 0.66–0.95; OR = 0.63, 95% CI = 0.49–0.83). In addition, the results also revealed that DPB1*1401 increased the anti-F antibody generation risk (OR = 4.86, 95% CI = 4.43–5.32), whereas DPA1*0201 decreased the anti-F antibody generation risk (OR = 0.58, 95% CI = 0.41–0.82) (Table 5). Moreover, the haplotypes analyses revealed three associated haplotypes: DPA1*0103-DPB1*0501 and DPA1*0103-DPB1*0201 were significantly associated with susceptibility to chronic HCV (OR = 1.60, 95% CI = 1.21–2.11; OR = 1.76, 95% CI = 1.39–2.25; respectively), DPA1*0202-DPB1*0501 and DPA1*0202-DPB1*0202 was associated with protective effects to chronic HCV infection (OR = 0.83, 95% CI = 0.71–0.97; OR = 0.67, 95% CI = 0.52–0.85). Furthermore, the haplotype DPA1*0103-DPB1*0201 and DPA1*0202-DPB1*0202, were significantly associated with susceptibility to anti-F antibody generation (OR = 1.38, 95% CI = 1.04–1.83; OR = 1.52, 95% CI = 1.04–2.22; respectively) (Table 6).

**Table 4:** Frequencies of haplotypes constituted with variant of rs3077 and rs9277534 between the two groups.

Haplotype ^#^	Anti-F Positive *n* = 516 (%)	Anti-F Negative *n* = 186 (%)	Healthy Controls *n* = 342 (%)	Cases ^†^ *vs.* Healthy Controls		Anti-F Positive *vs.* Anti-F Negative	
				OR (95% CI)	*p* *	OR (95% CI)	*p* *
CA	544 (52.7)	157 (42.2)	360 (52.6)	–	1.00 (reference)	–	1.00 (reference)
CG	165 (16.0)	48 (12.9)	127 (18.6)	0.86 (0.67–1.11)	0.249	0.99 (0.69–1.43)	0.966
TA	49 (4.7)	32 (8.6)	20 (2.9)	**2.08 (1.25–3.45)**	**0.004**	**0.44 (0.27–0.71)**	**0.001**
TG	274 (26.6)	135 (36.3)	177 (25.9)	1.19 (0.96–1.48)	0.123	**0.59 (0.45–0.77)**	**<0.001**

Abbreviation: OR, odds ratio; CI, confidence interval; **^#^** Haplotype was represented with the allele order of rs3077 and rs9277534; **^†^** Cases including the group of anti-F positive and anti-F negative; * The *p* values, OR, and 95% CI were calculated by Pearson Chi-Square test; Bold represent the *p* values were considered statistically significant.

### 2.2. Discussion

The mechanism of the development of chronic hepatitis C is complicated and it is necessary to look for some important clinical and genetic markers to help those who have a higher risk of developing chronic HCV. To our knowledge, the viral protein, immune response and interactions between the two sides certainly play a decisive role in the outcomes of HCV infection. HCV F protein is a derivative of the Core, expressed during HCV natural infection. Because the half-time of the HCV F protein is less than 10 min, we detected the anti-F antibody instead of the F protein itself in this study [29]. It is worth mentioning that the rate of F-seropositivity was increased along with the progression of the hepatitis C, in part because the virus produces a novel variant to adapt to the immune response of host [17]. In view of the pathogenicity of the HCV F protein which plays a special role in the progression of chronic hepatitis, research on the function of the HCV F protein in the immune response might explain a possible new vision of HCV evasion strategy or the propensity of HCV persistent infection. Meanwhile, several reports have described the association of *HLA* and non-*HLA* genes with chronic HCV or F protein, but few studies have been well-focused on the *HLA-DP* locus [7,17,19,20,21,30].

**Table 5:** Distribution of *HLA-DP* alleles among the group of anti-F negative patients, anti-F positive patients and healthy controls.

Alleles	Anti-F Positive *n* = 516 (%)	Anti-F Negative *n* = 186 (%)	Healthy Controls * *n* = 334 (%)	Cases ^†^ *vs.* Healthy Controls		Anti-F Positive *vs.* Anti-F Negative	
				OR (95% CI)	*P_adj_* **	OR (95% CI)	*P_adj_* **
*HLA-DPA1* alleles							
DPA1*0103	410 (39.7)	150 (40.3)	218 (32.6)	2.45 (2.0–3.0)	<0.001	0.98 (0.77–1.24)	NS
DPA1*0201	96 (9.3)	56 (15.1)	82 (12.3)	1.23 (0.93–1.65)	NS	0.58 (0.41–0.82)	0.006
DPA1*0202	526 (51.0)	166 (44.6)	368 (55.1)	0.79 (0.66–0.95)	0.042	1.29 (1.02–1.64)	NS
*HLA-DPB1* alleles							
DPB1*0201	218 (21.1)	73 (19.6)	91 (13.6)	1.66 (1.28–2.14)	<0.001	1.10 (0.82–1.48)	NS
DPB1*0202	124 (20.7)	29 (7.8)	108 (16.2)	0.63 (0.49–0.83)	0.016	1.62 (1.06–2.47)	NS
DPB1*0301	28 (2.7)	8 (2.2)	8 (1.2)	2.17 (1.00–4.70)	NS	1.27 (0.57–2.81)	NS
DPB1*0401	64 (6.2)	22 (5.9)	52 (7.8)	0.78 (0.54–1.11)	NS	1.05 (0.64–1.73)	NS
DPB1*0402	160 (15.5)	57 (15.3)	124 (18.6)	0.80 (0.63–1.02)	NS	1.01 (0.73–1.41)	NS
DPB1*0501	314 (30.4)	121 (32.5)	210 (31.4)	0.98 (0.80–1.19)	NS	0.91 (0.70–1.17)	NS
DPB1*0601	2 (0.2)	2 (0.5)	6 (0.9)	0.32 (0.09–1.12)	NS	0.26 (0.04–1.88)	NS
DPB1*0901	12 (1.2)	7 (1.9)	10 (1.5)	0.90 (0.42–1.95)	NS	0.61 (0.24–1.57)	NS)
DPB1*1301	20 (1.9)	4 (1.1)	8 (1.2)	1.44 (0.64–3.21)	NS	1.82 (0.62–5.36)	NS
DPB1*1401	0 (0)	8 (2.2)	12 (1.8)	0.31 (0.13–0.77)	NS	4.86 (4.43–5.32)	<0.001
DPB1*1701	16 (1.6)	11(3.0)	12 (1.8)	1.07 (0.54–2.13)	NS	0.52 (0.24–1.12)	NS
DPB1*1901	20 (1.9)	7 (1.9)	7 (1.0)	1.85 (0.80–4.27)	NS	1.03 (0.43–2.46)	NS
DPB1*1001	18 (1.7)	10 (2.7)	8 (1.2)	1.68 (0.76–3.70)	NS	0.64 (0.29–1.41)	NS
DPB1*1601	8 (0.8)	5 (1.3)	3 (0.4)	2.07 (0.59–7.30)	NS	0.42 (0.14–1.29)	NS
DPB1*2101	16 (1.6)	3 (0.8)	3 (0.4)	3.04 (0.90–10.31)	NS	1.94 (0.56–6.69)	NS
DPB1*3901	2 (0.2)	0 (0)	2 (0.3)	0.48 (0.07–3.38)	NS	1.36 (1.32–1.41)	NS

* Healthy controls: 8 samples were excluded because of genotyping failure; **^†^** Cases including the group of anti-F positive and anti-F negative; ** The *p* value were adjusted (*p*_adj_) for multiple alleles by applying the Bonferroni correction of multiplying the *p* value by the total number of alleles tested in each locus.

**Table 6:** Distribution of *HLA-DPA1-HLA-DPB1* haplotypes among the group of anti-F negative patients, anti-F positive patients and healthy controls.

Haplotype	Anti-F Positive (%)	Anti-F Negative (%)	Healthy Controls (%)	Cases ^†^ *vs.* Healthy Controls		Anti-F Positive *vs.* Anti-F Negative	
				OR (95% CI) *	*p* *	OR (95% CI) *	*p* *
DPB1*0402-DPA1*0103	6.8	8.6	7.9	0.91 (0.71–1.16)	0.440	0.77 (0.57–1.05)	0.101
DPB1*0402-DPA1*0202	7.5	5.6	8.2	0.84 (0.66–1.07)	0.149	1.35 (0.95–1.92)	0.096
DPB1*0501-DPA1*0103	7.8	9.1	5.2	**1.60 (1.21–2.11)**	**0.001**	0.84 (0.62–1.12)	0.235
DPB1*0501-DPA1*0201	1.6	1.3	1.6	0.91 (0.54–1.53)	0.713	1.16 (0.57–2.36)	0.691
DPB1*0501-DPA1*0202	21.1	21.6	24.6	**0.83 (0.71–0.97)**	**0.017**	0.97 (0.79–1.19)	0.768
DPB1*0401-DPA1*0103	3.2	4.3	4.6	0.74 (0.54–1.03)	0.072	0.74 (0.48–1.13)	0.160
DPB1*0401-DPA1*0202	3.0	2.0	2.7	1.02 (0.68–1.52)	0.930	1.51 (0.85–2.66)	0.157
DPB1*0201-DPA1*0103	12.4	9.3	6.9	**1.76 (1.39–2.25)**	**<0.001**	**1.38 (1.04–1.83)**	**0.024**
DPB1*0201-DPA1*0202	6.7	5.0	4.8	1.30 (0.97–1.74)	0.078	1.37 (0.94–1.99)	0.098
DPB1*0202-DPA1*0202	7.0	4.7	9.3	**0.67 (0.52–0.85)**	**0.001**	**1.52 (1.04–2.22)**	**0.030**

Abbreviation: OR, odds ratio; CI, confidence interval; **^†^** Cases including the group of anti-F positive and anti-F negative; * The *p* values, OR, and 95% CI were calculated by Person Chi-Square test; Bold represent the *p* values were considered statistically significant.

In this study, we found that rs3077 in the *DPA1* locus significantly increased the risk of chronic HCV infection. This means that persons who carry the minor T alleles of the rs3077 have higher risk of chronic HCV than those subjects with wild type allele C. This finding may provide a novel way to understand the complex mechanism of the formation of chronic hepatitis C. In addition, we also found that the rs3077 in the *DPA1* locus was a potentially protective marker of anti-F antibody generation in the Chinese population. Specific antibodies and T-cell responses against HCV F protein were detected in the sera of HCV-infected patients and F protein generation after viral exposure varies enormously among individuals. This study also found that the number of platelets were reduced with HCV F protein expression. The low platelet secretion by the bone marrow is an important causative factor of thrombocytopenia in liver cirrhosis, and the relationship between functional liver mass and peripheral platelet count has been well established [31,32,33]. Meanwhile, the relationship between HCV F protein and liver fibrosis stage also verify the conclusion. Apart from these, several reports have found that the production of HCV F protein might be influenced by the treatment (PEG-IFNα/RBV) and be associated with a sustained virological response (SVR) in hepatitis C patients. Branch *et al.* reported that chronic HCV patients with IFN therapy have a higher proportion of HCV F-seropositive than those not [34]. Deyong *et al.* found that the rate of F-seronegative patients who achieved a SVR was higher than those patients with F-seropositive [28]. Moreover, our group previously also demonstrated that the HCV F protein may inhibit peripheral blood mononuclear cells IFN-α secretion by regulating the production of interleukin (IL)-10, and may also contribute to apoptosis in plasmacytoid dendritic cells [35]. These findings all indicate that the presence of F antibodies may be an indicator that predicts the efficacy of antiviral treatment in patients with HCV infection. In addition, the F protein could induce type 2 T helper (Th2) cell biased cytokine response and IL-6 secretion in HCC patients [15,17]. The imbalance of Th1/Th2 responses in patients with HCV infection may contribute to the disease progression, and further, IL-6 is a proinflammatory cytokine for which increased levels can result in chronic liver inflammation, and even progression to HCC. Therefore, based on these points, we suspect that with continuous development of the disease and the increased proportion of F-seropositive under the pressure of host immune response and medications, the F protein may be a novel product to adapt to the host environment and prompt virus survival in the host. Thus, the variant alleles of two SNPs decrease the risk of anti-F antibody generation, and may also decrease the risk of HCV F protein generation after HCV infection to reduce the pathogenicity of the antigen. On the other hand, other studies also considered that the increased expression of F protein under the virus-host struggle, compared with Core expression and function, would relatively decrease virus pathogenicity and play a protective role in the host. The reasons are as follows: firstly, HCV F is a double-frame shift product of the HCV core gene and the discovery of the F protein certainly challenges various biological functions attributed to Core. The F protein, not a substitute for Core, is not involved in HCV replication and regulation of some proto-oncogene or anti-oncogenes, including c-myc, p53 [36,37]. Secondly, the level of Core expression was also downregulated by the intracellular synthesized F protein, which has interrelated HCV RNA secondary stem-loop structure [36,38]. Our team previously found that the HCV F protein can induce the protective T-cell-mediated immune response [15]. We reveal here for the first time, the risk factors (variants in *HLA-DPA1* and *HLA-DPB1*) associated with chronic HCV infection and anti-F antibody generation. In order to better illustrate the issues raised above, we conducted the haplotype analysis by logistic regression and considered that those persons with a T–A haplotype may have a higher risk of HCV persistent infection than those persons with a C–A haplotype, whereas people with T–A or T–G may have a lower risk of F protein production when compared with the most frequent haplotype.

Furthermore, according to the report rs3077 and rs9277534, located in the 3'-UTR of *HLA-DPA1* and *HLA-DPB1*, respectively, were not only identified as the SNPs with the highest significance level associated with disease progression, but also reported to be strongly associated with decreased mRNA expression of *HLA-DPA1* and *HLA-DPB1* [23,27,39]. Based on the web-based SNP analysis tool [40], we explored the S ariants in the gene affect the binding site of some microRNA (miRNA), affecting the stability and translation of mRNA. According to the report, the variants of the miRNA-binding site are likely to destroy the interaction between the miRNA and the target gene, and result in the deregulation of target gene expression [41]. Further, it is reported that the miRNA is also significantly associated with the cleavage and release of the HCV genome RNA [42]. Moreover, some reports also revealed that the variation in the 3’-UTR of the genes may also affect the 3' end formation of pre-messenger RNA, messenger RNA secondary structure and related genes’ methylation [43,44,45,46]. Taken together, the above evidence suggests that the variants in the *HLA-DP* might lead to lower levels of *HLA-DP* molecular expression on target cells surfaces and cause less effective processing and presenting of viral antigen to CD4^+^ T helper cells, resulting in an impaired immune response to the virus infection. Moreover, the variants in the *HLA-DP* locus may also damage the interactions between the *HLA* molecular and rested antigen-presenting cells, including cytotoxic T lymphocytes, B lymphocytes, dendritic cells (DC), and natural killer (NK) cells, and affect the innate immune response and adaptive immune response [47]. Specially, according to the report of Png *et al.*, the variants in *HLA-DR* (rs3135363), *HLA-DPB1* (rs9277542) and *HLA* class III (rs9267665) loci strongly influenced the antibody titers after HBV vaccination [48]. This is partly because the abnormal or lower level of the *HLA* molecule on the surface of CD4^+^ T affects B lymphocyte activation to secrete the neutralizing antibodies [49]. Based on this point, we also suspected the variants in *HLA-DP* may also influence the anti-F antibody titers. However, this hypothesis needs to be validated by experiments in further functional studies.

*HLAs* play a vital role in humoral and cellular immunity, and its allele polymorphisms have been reported to be involved in the immune responses to viruses, including HIV, HBV and HCV [21,23,50]. HLA-DRB1*11, HLA-DQB1*0301 and HLA-DRB1*07 were found to be associated with HCV clearance or persistence, and HLA-DPA1*0103, HLA-DPB1*0402 and HLA-DPB1*0501 were found to be candidate predictive factors for antibody production after HBV vaccination [51,52,53]. Moreover, Bain *et al.* previously identified a specific CD8^+^ memory T cell response for *HLA-A2* and/or *HLA-B7* predicted epitopes derived from the HCV F protein in patients with HCV natural infection [16]. Based on this evidence, we considered that the *HLA* II alleles polymorphism may also have significant association with chronic HCV infection or anti-F antibody generation, and the relationship between these parts will be beneficial in predicting the outcomes of disease or therapeutic regimens. Moreover, *HLA-DP* genes, highly polymorphic in exon 2, encode antigen-binding sites and are receptors for processed peptides derived predominantly from membrane and extracellular proteins. So the polymorphism of the *HLA-DP* alleles may lead to amino acids substitutions in antigen-binding sites or change the ligand–receptor binding affinity leading to a weakening of the immune response to HCV invasion. In our analysis, we revealed susceptibility or protective haplotype to chronic HCV infection and anti-F antibody generation. *HLA-DPs* belong to the *HLA* class II molecules that form heterodimers on the cell surface and present antigens to CD4^+^ T cells. The strong response of helper T cells to HCV has been considered a vital way to resolve acute HCV infection [54]. The haplotype analysis suggested that chronic HCV and anti-F antibody generation were associated with haplotypes containing the *HLA-DPA1* and *HLA-DPB1* genes, so firstly, we considered that polymorphism of *HLA-DP* molecules would affect the ability of effective epitope presentation on immune cells and result in weak immune response to virus invasion. Gao *et al.* reported that at least three peptides within HCV F protein were identified as HLA-DRB1*01 or HLA-DP*0401 presenting epitopes in humanized mouse models by the splenocyte proliferation assay [55]. Secondly, we also inferred that the different *HLA-DP* alleles may exhibit different protein expression level or antigen receptor repertoires to influence the ability of HCV antigen presentation. Several studies might support this hypothesis, that the *DPA1* residues 9, 11, 35, 55, 56, 69 and 84–87 and a single protein substitute (K to E at position 69) of *DPB1* influence T cell allorecognition or peptide binding, and the impact of *HLA-DP* to HBV may depend on different expression levels rather than differences in peptide presentation [27,56,57,58]. However, this is just a deduction, more studies are warranted to clarify this problem. Interestingly, we also found that DPB1*0202-DPA1*0202 not only protects from chronic HCV infection but also is associated with anti-F antibody generation. This result may verify that the F protein acts as a double-edged sword in the pathogenesis of chronic HCV, because, on the one hand, the protective T-cell-mediated immune response specific to F protein may lead to viral clearance and have anti-tumor effects; on the other hand, F protein may be a virulence factor by helping HCV to survive in the adverse conditions, with molecular events that likely contribute to viral persistence at a low level of replication [15]. Compared with healthy subjects, we think that the haplotype DPB1*0202-DPA1*0202 may protect from chronic HCV infection. Once a person is infected with HCV, the expression of the F protein not only decreases the generation of HCV core protein, but also relatively reduces virus pathogenicity. On the other side, F protein may be a new causative factor in regulation of the host environment and prompting virus survival in the host. However, it is remain unclear that how the F protein balances between positive and negative effects.

Our study had several strengths: first of all, subjects with HCV infection came from a systematic screening of HBV and HIV markers in a population-based study conducted in Jiangsu Province which may have reduced potential selection bias. Secondly, the subjects with or without anti-F antibody were selected from people with chronic HCV infection, decreasing the bias of different pathological states. Thirdly, the study was conducted with novel pathogenic antigens and thus provides a new perspective to understand the mechanism of HCV evasion strategy. However, the number of patients in our study was relatively small, and the statistical power of the research was limited. Further, many important clinical data values, including α-fetoprotein level, were not included in this study. Also, larger sample studies with ethnically diverse populations are warranted. Moreover, *IL-28B* genotypes (rs8099917, rs12979860) influence the SVR and spontaneous HCV clearance, partly because the favorable *IL-28B* genotypes influence the production of IFNλs in hepatitis C [9,59]. Whether there are some relationships among *IL-28B* genotypes, *HLA* alleles and HCV F protein remains to be further elucidated.

## 3. Experimental Section

### 3.1. Study Subjects

A total of 1044 subject were enrolled between June 2012 and December 2012 at the Jurong and Danyang city, Jiangsu Province, China, including 702 chronic HCV infection cases and 342 normal controls. The exclusion criteria of the cases included the HBV or HIV infection, other types of liver diseases, or previous interferon and/or ribavirin therapy. Subsequently, all subjects were grouped into three different groups for analysis: Healthy controls included 342 normal subjects, who were anti-HCV seronegative; anti-F negative composed of 186 F-seronegative chronic HCV infection cases, who were anti-HCV seropositive, HCV RNA seropositive and F-seronegative; anti-F positive consisted of 516 F-seropositive chronic HCV infection cases, who were anti-HCV seropositive, HCV RNA seropositive and F-seropositive.

Each subject signed the informed consent form before being recruited in the study, and was scheduled for a face-to-face interview by trained physicians and nurses. The interview included a structured questionnaire with information on demographics and related environmental exposure history. After the interview, about 5 mL of venous blood was collected from each subject for further genotyping assays, blood test, liver function and virus detection. The stage of liver fibrosis was measure by transient elastography (FibroScan^®^, Echosens, Paris, France) [60]. The ethics approval of the study (January 2014–December 2015) was acquired from the Human Investigation Committee of the Huadong Research Institute for Medicine and Biotechnics.

### 3.2. Virological Tests

Anti-HCV antibodies were tested by the third generation HCV enzyme immunoassays (KHB, Shanghai, China). HCV RNA was extracted from serum using Trizol reagent (Trizol LS Reagent, Life Technologies, Rockville, MD, USA) and detected by polymerase chain reaction (Realchip Biotechnology Co., Ltd., Ningbo, China) according to the manufacturer’s instructions. The reaction products were detected in a 2% agarose gel (Biowest Agarose, Hong Kong, China) stained with Gel stain (Beijing Transgen Biotech Co., Ltd., Beijing, China). The HCV F protein was expressed and purified by our group team [15]. The detection of anti-F antibodies was performed by indirect ELISA according to the report described by Kong *et al.* [15]. The subject with serum detected anti-F antibody was named F-seropositive sample, otherwise, named F-seronegative sample.

### 3.3. Genotyping Assays

Genomic DNA was extracted from leucocytes of venous blood samples by sodium dodecyl sulfate lysis and protease K digestion followed by phenol-chloroform extraction and ethanol precipitation. SNPs rs3077 and rs9277534 were genotyped by the TaqMan allelic discrimination assay on an ABI 7900HT Fast Real-Time PCR system (Applied Biosystems, Foster City, CA, USA). The rs3077 C/T (major/minor alleles in Asians) in this study corresponds to the G/A alleles, respectively, on the reverse strand in Kamatani *et al.* [23]. The information of primers and allele-specific fluorogenic probes of rs3077 and rs9277534 are shown in Table 7. In addition, genotyping was performed without the knowledge of the subjects’ case or control status, and two negative controls (water) included in each 384-well plate were used for quality control. At last, the results of genotyping were determined by using SDS 2.3 Allelic Discrimination Software (Applied Biosystems, Foster City, CA, USA). The concordant result of the each SNP were 100% for the 10% samples randomly selected to repeated assays.

**Table 7:** The information on primers and allele-specific fluorogenic probes for the two single nucleotide polymorphisms (SNPs).

SNPs	Name	Sequence (5'–3')
rs3077	Primes sense	TCAGCTTTTCTTCTCACTTCATGTG
	Primes antisense	GAGCTTGAAGGGTCAGCAATTC
	Probes allele C	FAM-AAACTACCCCAGTGGC-MGB
	Probes allele T	HEX-AAACTACTCCAGTGGCT-MGB
rs9277534	Primes sense	CCAAATCAAGTTTAGTGCCCTCAT
	Primes antisense	GCAGTCTGCTCACCATTGAATAGT
	Probes allele A	FAM-CTCAGACCACTATTC-MGB
	Probes allele G	HEX-TCAGACCGCTATTC-MGB

### 3.4. HLA-DPA1 and HLA-DPB1 Genotyping

We analyzed *HLA-DPA1* and *HLA-DPB1* genotypes using 702 cases and 342 controls. Exon 2 of the *HLA-DPA1* and *HLA-DPB1* genes were amplified and determined by sequencing-based typing (SBT) by Huada Gene (Shanghai, China). *HLA-DPA1* and *-DPB1* alleles were validated based on the *IMGT/HLA* database [61].

### 3.5. Statistical Analysis

The Hardy-Weinberg equilibrium (HWE) was tested by a goodness-of-fit χ^2^ test to compare the observed genotype frequencies to the expected ones among the three group subjects. Distributions differences of general demographic characteristics, clinical, selected variables and frequencies of genotypes between the cases and the controls were evaluated by using the Student’s *t* test, one-way analysis of variance and the χ^2^. Logistic regression analyses with adjustment for age, sex and/or HCV genotype were used to test the association between genotypes and chronic HCV infection risk or anti-F antibody generation, to estimate odds ratios (ORs) and 95% confidence intervals (CIs). The Cochran-Armitage test was used for trend analysis. Haploview was employed to analyze linkage disequilibrium (LD) parameters (*i.e.*, *D*’ and *r*^2^). PHASE v2.1 software was used to estimate the haplotype for each subject based on the observed genotypes [62]. ORs of each haplotype were calculated relative to the major haplotype. *HLA-DPA1* and *HLA-DPB1* allele frequencies and *DPA1*–*DPB1* haplotype frequencies were calculated by direct counting. To assess the association of each *HLA* allele (*HLA-DPA1* and *HLA-DPB1*) and *DPA1*–*DPB1* haplotypes, we used χ^2^ test or Fisher’s exact test. All the statistical analysis were carried out using SPSS software version 17.0 (SPSS Inc., Chicago, IL, USA). The values of *p* were two-sided, and less than 0.05 was considered the level of statistical significance.

## 4. Conclusions

In summary, we report for the first time that the variant (rs3077) and alleles of *HLA-DP* loci were associated with chronic HCV infection or anti-F antibody generation. We also identified the chronic HCV risk/protective haplotypes and the anti-F antibody generation risk haplotypes. Our study suggests that *HLA-DPA1/B1* loci are candidate susceptibility regions that have some marker SNPs for both chronic HCV infection and F protein generation in the Han Chinese population. These results might provide an insight into the molecular mechanisms of disease progression during HCV chronic infection. Although our study indicates variants at *HLA-DP* were significantly associated with chronic HCV infection and anti-F antibody generation, biological function analysis remains to be elucidated.
